# In-culture coronary stenting in an *ex vivo* vascular bioreactor

**DOI:** 10.3389/fcvm.2025.1565674

**Published:** 2025-07-31

**Authors:** F. Razzi, J. Bobi, M. Stijnen, J. H. van Esch, D. J. Duncker, V. van Steijn, H. M. M. van Beusekom

**Affiliations:** ^1^ Division of Experimental Cardiology, Cardiology Department, Erasmus MC University Medical Center, Rotterdam, Netherlands; ^2^ Department of Chemical Engineering, Faculty of Applied Sciences, TU Delft, Delft, Netherlands; ^3^ LifeTec Group BV, Eindhoven, Netherlands

**Keywords:** vascular bioreactor, stent implantation, coronary artery, ex vivo models, preclinical research, cardiovascular diseases

## Abstract

**Background:**

*Ex vivo* vascular bioreactors that enable interventions in arteries from slaughterhouse surplus hearts present valuable alternatives to animal models to test cardiovascular stents. However, the knowledge for stent implantation during *ex vivo* culture in slaughterhouse coronary arteries is limited. The objective of the study is two-fold: first, to determine culture settings, the time point and optimal conditions for in-culture stent implantation using surplus right coronary arteries (RCAs) from swine with known *in vivo* RCA diameters; and second, to implement the gained insights to culture and stent RCAs obtained from slaughterhouse hearts (unknown *in vivo* diameter).

**Methods:**

Swine RCAs were mounted, cultured and stented in an *ex vivo* vascular bioreactor (VABIO) under conditions of flow and pressure. The bioreactor culture and stenting protocols were optimized using a step wise approach. In Step 1, the RCAs dissected from in-house swine hearts, with known diameters, were cultured until endothelialized as the ideal time point for stenting, and the stent implantation procedure was optimized. In Step 2, the successful *ex vivo* stent implantation procedure was repeated in slaughterhouse RCAs. Structural changes of the RCAs were assessed by ultrasound imaging during culture. The morphology of the RCAs at the end of culture was assessed by histology.

**Results:**

The RCAs adapted to the *ex vivo* environment, stabilizing their diameter in the range of the *in vivo* diameter after day 3, which was selected as the earliest time point for stenting. Because stent implantations caused mural dissections in the RCAs, visible with ultrasound imaging and confirmed by histology, we developed an external support for the RCA. This was found to be critical for better physiological intravascular pressures and to minimize dissections upon stent implantation. Finally, the stent implantation procedure was successfully replicated in slaughterhouse arteries.

**Conclusions:**

Our study demonstrates the feasibility of in-culture *ex vivo* stent implantation in the VABIO, providing important requirements and useful insights for *in vivo* mimicking stent implantation, for future investigations in slaughterhouse arteries.

## 1 Introduction

Cardiovascular disease is the leading cause of death globally, with coronary artery disease (CAD) accounting for nearly half of the victims (1). CAD is mainly caused by the development of atherosclerosis and manifests itself in the narrowing of the artery lumen (2). The preferred treatment for symptomatic CAD is stent implantation. Despite the increased procedural success and improved long-term outcomes, some patients still present with complications like in-stent restenosis, neo-atherosclerosis and late stent thrombosis, requiring secondary interventions (3). The development of new stents that minimize these complications is still needed.

Swine coronary arteries are considered as the gold-standard model for the assessment of safety and efficacy due to their close resemblance to human arteries (4). However, *in vivo* models present some limitations due to high costs and ethical concerns as well as due to the difficulty to repeatedly monitor treated arteries over time (5). As an alternative to *in vivo* animal and *in vitro* cell culture models, *ex vivo* vascular bioreactors become more relevant for studying vascular responses to biomechanical and biochemical factors as they can provide the arteries with physiologically relevant conditions while the arterial three-dimensional structure is maintained (6, 7).

*Ex vivo* vascular bioreactors using arteries from slaughterhouse organs could provide an excellent opportunity for testing arterial interventions while reducing the number of animals and associated costs in the preclinical phase if relevant physiological conditions can be mimicked (6). To date, such *ex vivo* interventions have been mainly performed on porcine carotid and coronary arteries from slaughterhouse material, performed *ex vivo*, before starting the *ex vivo* culture (5, 8, 9), rather than in-culture. Hence, this strategy does not allow controlled adaptation of the artery from the *in vivo* to the *ex vivo* environment and it remains unclear whether the artery is cultured under relevant conditions in terms of its *in vivo* diameter. As a consequence, parameters relevant for the intervention such as the size of the stent and balloon are not based on the *in vivo* features of the artery which may affect the vascular response to the interventions. This raises the question “how long does it take for the *ex vivo* artery to adapt to culture conditions and regain its *in vivo* diameter?”. The answer to this question is instrumental for tuning the bioreactor to best mimic *in vivo* hemodynamic parameters and to allow in-culture arterial interventions.

We therefore designed a pragmatic two-step study for *ex vivo* culturing of coronary arteries obtained from slaughterhouse hearts in a bioreactor with the capability to perform ultrasound (US) imaging assisted stent implantation during the culture. The first aim was to determine the ideal time point for *ex vivo* stent implantation based on *in vivo* coronary artery diameters. For this purpose, we cultured surplus right coronary arteries (RCAs) obtained from in-house swine for which we measured the *in vivo* diameter by angiography. The RCA was chosen as it is relatively long with a stable diameter, is subjected to less forces during heart contraction and shows a more stable flow pattern than the left coronary system during the heart cycle. This makes it easier to mimic relevant physiologic conditions in the bioreactor. The second aim was to determine the ideal circumstances to perform *ex vivo* coronary stent implantation and maintain the culture 2 to 5 days after stenting, common time points used *in vivo* to assess early vascular reactions (10). The final aim was to use the gained insights on in-house swine to culture and stent RCAs obtained from slaughterhouse hearts in the bioreactor with *in vivo*-mimicking conditions.

## 2 Materials and methods

### 2.1 Study design

**Figure 1:**
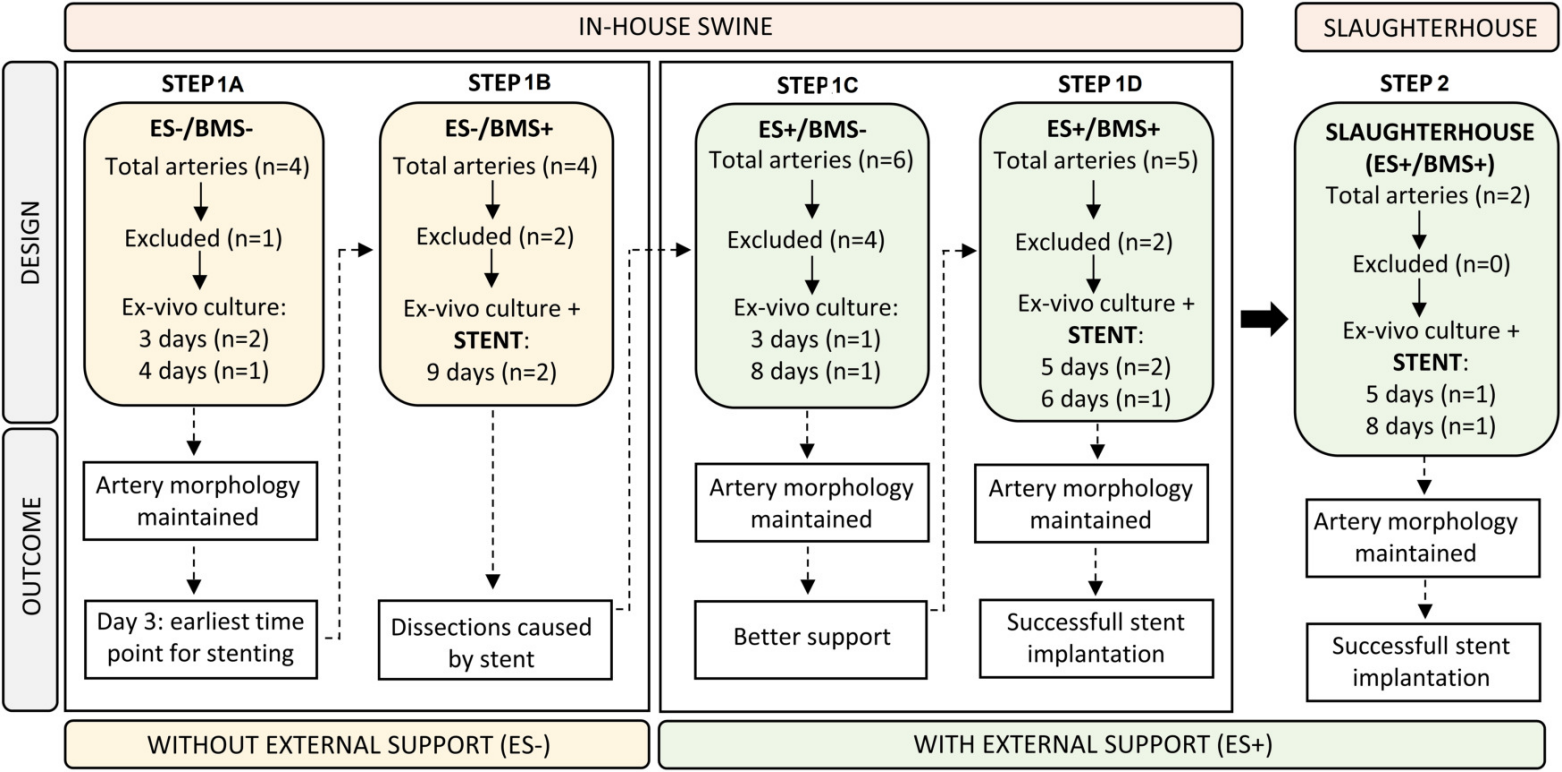
Flow chart illustrating the study design. The design of each step (top half) was based on the outcome (bottom half) of the preceding step. Step 1A and B contain RCAs that were *ex vivo* cultured without the external support (ES-), while RCAs from Step 1C and 1D were cultured with the addition of the external support (ES+). Insights on in-house swine from Steps 1A–D were used to culture and stent slaughterhouse swine in Step 2. ES− = without external support; ES + = with external support; BMS− = without stent; BMS+ = with stent.

In Step 1, addressing aims 1 and 2, we used coronary arteries from in-house swine to allow a direct comparison between *in vivo* and *ex vivo* RCA diameters to determine how long it takes for an RCA to stabilize in the bioreactor and, regain the *in vivo* diameter of the RCA. Stents were implanted in the cultured RCAs but implantation resulted in dissections of the artery wall. To give support to the artery and to optimize the stent implantation procedure, the RCAs were then cultured with an external support made of agarose as a replacement of the *in vivo* surrounding tissue of the RCA before stent implantation. In step 2, addressing aim 3, we used the optimized procedures developed in step 1, to culture and stent RCAs obtained from slaughterhouse tissue as the envisioned waste material. The description of each step is explained more extensively in the Results section (Figure 1).

### 2.2 Bioreactor development

**Figure 2:**
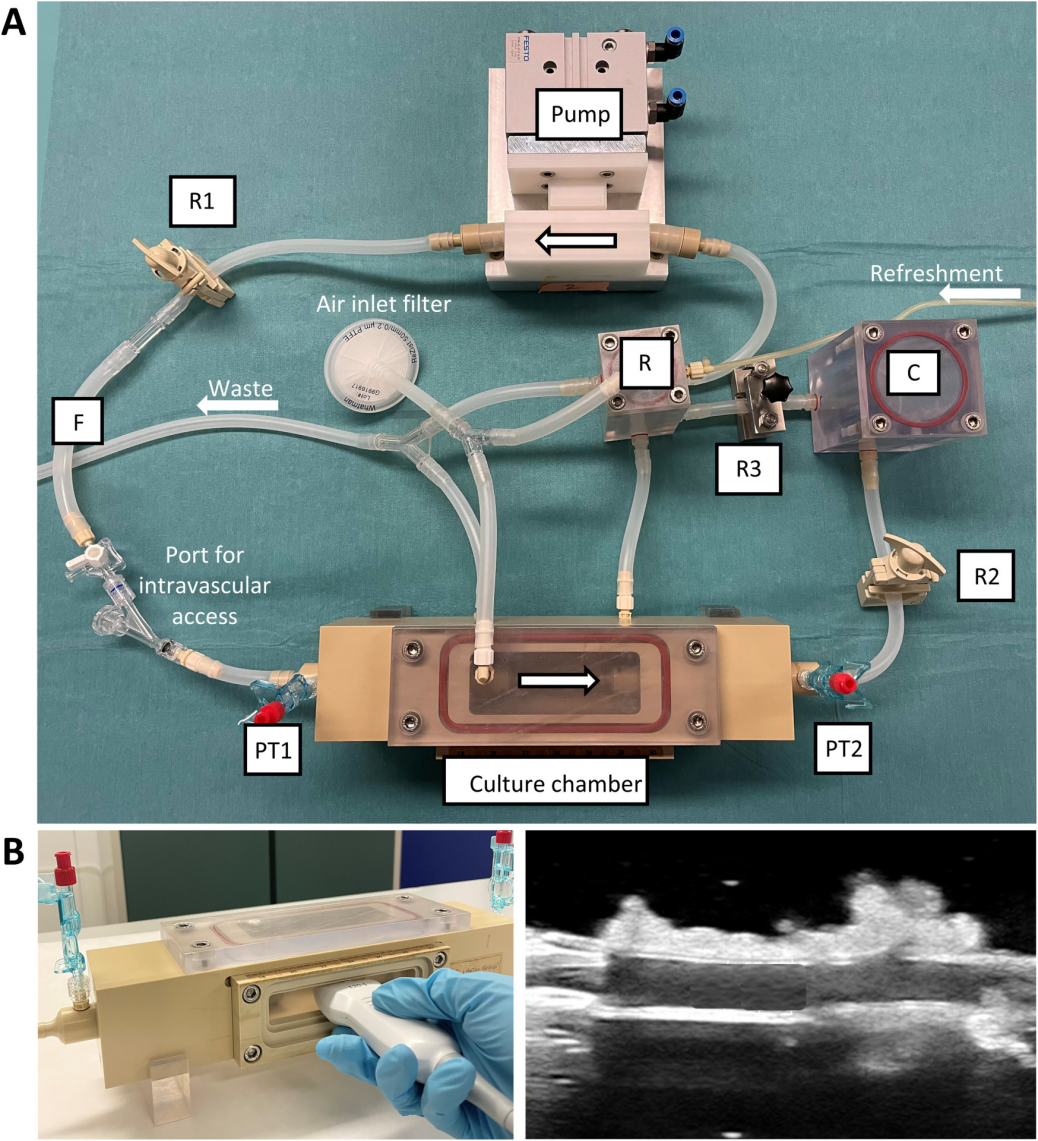
*Ex vivo* vascular bioreactor system. **(A)** Photograph of the circulation system showing the components. **(B)** The culture chamber presents a transparent silicone window through which US imaging can be performed. R = reservoir; R1, R2, R3 = resistances; F = flow sensor; PT1, PT2 = pressure transducers; C = compliance chamber.

We optimized the *ex vivo* vascular bioreactor developed before (6) (VABIO, LifeTec Group, The Netherlands) to the cultivation and stenting of RCAs. The scheme of the bioreactor is presented in Figure 2A. A circulation pump was used to distribute the liquid throughout the bioreactor. The liquid entered the pump from a reservoir (Figure 2A, “R”) to prevent air from entering the circuit. To tune the pressure and flow produced by the circulation pump and to mimic the *in vivo* pressure inside the vessel, we used a compliant tube and a resistor (Figure 2A, “R1”) and a set of two variable resistances (Figure 2A, “R2” and “R3”) and a compliance chamber (Figure 2A, “C”) (Windkessel model).

The artery was positioned in the circuit by mounting it in the culture chamber (Figure 2A). The culture chamber featured an optically transparent window that allowed for the measurement of the inner diameter of the artery by means of US imaging (Figure 2B). The flow rate was measured between the resistor and the culture chamber (Figure 2A, “F”). The pressure was measured upstream (PT1) and downstream (PT2) of the culture chamber. Oxygen levels of around 95% were obtained by sterile gas exchange between the culture medium with the external environment in the incubator via passive diffusion through 0.22 µm filters (Figure 2A, “Air inlet filter”). An external medium refreshment line was connected to the reservoir to allow for the introduction of fresh medium through a roller pump. The medium excess (waste line) was collected from the reservoir and the culture chamber in a waste bottle via drainage of the liquid.

### 2.3 *In vivo* angiography

For Step 1, we used hearts (surplus material) (*n* = 19) from Yorkshire-Landrace female swine (40–50 kg) originally bred for slaughter but undergoing brain vascular interventions in our department. This allowed us to measure the *in vivo* diameter of the RCAs before the *ex vivo* culture. The animals were part of a study approved by the Animal Ethics Committee of the Erasmus University Medical Center Rotterdam, The Netherlands (AVD1010020198546). The RCAs of the animals were not targeted during the experiments and unlikely influenced by the brain vascular interventions. At the end of the experiments, right before sacrifice, *in vivo* angiography of the RCAs was performed without using any vasodilators (restrictions due to experimental set-up). Heparin (10,000 IU) was given to prevent clotting just prior to sacrifice, animals were sacrificed under deep anesthesia, and the hearts were collected.

The *in vivo* diameter of the RCAs was measured using dedicated software (Caas MR Solutions version 8.1.1, Pie Medical Imaging). We note that swine coronary arteries are prone to vasoconstriction when cannulated by catheters or injected with room-temperature contrast. Based on a pilot measurement (data not shown), we observed that the addition of intracoronary nitroglycerine (200 µg), normally used in clinical practice, increases the RCA diameter by 15%–20%. Based on this insight, we corrected the measured *in vivo* diameters by adding 15%.

### 2.4 Tissue harvesting and handling

For step 1 (in-house swine), hearts were excised, placed in DMEM with 2% penicillin-streptomycin (antibiotic) and 0.6% amphotericin B (antimycotic), and stored at 4°C overnight prior to tissue preparation. For step 2 (slaughterhouse tissue), swine hearts (*n* = 2) were collected from a local slaughterhouse (Westfort, IJsselstein, The Netherlands). To preserve their functionality and reduce the risk of infection, the hearts were placed in cold sterile Krebs buffer with the addition of 2% penicillin-streptomycin and 0.6% amphotericin B. After collection, hearts were transported to the laboratory while kept at 4°C. The RCAs were dissected within 1–3 h after collection of the hearts.

### 2.5 Tissue preparation

The RCAs (5–6 cm length) were dissected free in a sterile environment using surgical tools while being flushed with cold DMEM. The perivascular tissue was removed to visualize and close the coronary side branches with sutures to avoid leakage and ensure maintenance of flow and pressure throughout the entire *ex vivo* culture. Arteries were mounted in the bioreactor connectors, adding papaverine (0.5 mg in 30 ml of DMEM) as a vasorelaxant. To check for unwanted leakages, arteries were gently pressurized with culture medium.

### 2.6 Culture medium

The culture medium used to perfuse the arteries in the bioreactor replicates the most relevant characteristics of blood such as nutrients and growth factor content, oncotic pressure and viscosity. It was prepared as described before (6). In short, it comprises of DMEM with 1 g/L D-glucose supplemented with 50 µM 2-mercaptoethanol, 1% antibiotic penicillin-streptomycin, 0.75 ng/ml antimycotic amphotericin B, 10% fetal bovine serum, 2.5 ng/ml vascular endothelial growth factor and sterile Xanthan gum (0.66 g/L) for viscosity.

### 2.7 Culture protocol

Before the experiments, the clean bioreactor was assembled and autoclaved at 121°C for 1 h. The autoclaved bioreactor was then placed in a biosafety cabinet. For the cultures without external support, the bioreactor was filled with 200 ml of medium to check for leakages and to remove air from the tubes. After surgical dissection of the RCAs from the hearts, the RCAs were mounted in the culture chamber (filled with medium) and longitudinally aligned to the axis of the chamber, preventing excessive stretch. The system was then placed in an incubator at 38°C [to mimic swine body temperature (37.5–39.5°C)], at 5% CO_2%_ and 100% humid atmosphere, and connected to the circulation pump. RCAs contracted during transport and surgical dissection and, in order to acclimatize the RCAs to the *ex vivo* environment and to minimize the damage, the pressure and flow were gradually increased during the first 5 h of culture. After this initial phase, the artery inner diameter was measured and the flow rate was adjusted, following the increase in artery diameter, to apply a physiological peak shear stress to the endothelium, in the range of 0.6 to 2.1 Pa (11). The Poiseuille equation for laminar flow was used:

$$\mathrm{Qpeak}=\;\frac{\pi \tau D^{3}}{32\mu }$$

with Qpeak = peak flow rate, *τ* = peak shear stress, D = vessel inner diameter, µ = medium viscosity. The pressure was also adjusted by regulation of the variable resistances R1, R2 and R3, and by adjusting the pump speed. To keep nutrient and waste product levels constant during culture, 50 ml of fresh medium was provided every day. At the end of the cultures, the RCAs were removed from the culture chamber and the lumen was gently flushed from proximal to distal with 4% buffered formaldehyde. The RCAs were fixed in 4% buffered formaldehyde for at least 48 h for further histological analysis.

### 2.8 Data acquisition

During the *ex vivo* coronary artery culture, the pressure, flow rate and diameter were monitored daily. Pressure and flow signals were acquired using a PC with custom LabView-based software through a breakout box and a data acquisition system, as described before (6). The intravascular pressure curves as presented in this work are the average of PT1 and PT2. The systolic (peak), diastolic (lowest) and mean arterial pressure (SAP, DAP and MAP, respectively) were calculated from the averaged pressure curves.

The flow rate was measured by an ultrasonic flow sensor (SONOFLOW CO.55 V2.0, Sonotec) placed before the culture chamber. In order to assess cultured blood vessel morphology and diameter, US imaging was performed using an US machine equipped with a linear probe (L20-5, Zonare Medical Systems). The inner arterial diameter was calculated from the average diameter of three segments: proximal, middle and distal.

### 2.9 Stent implantation

**Figure 3:**
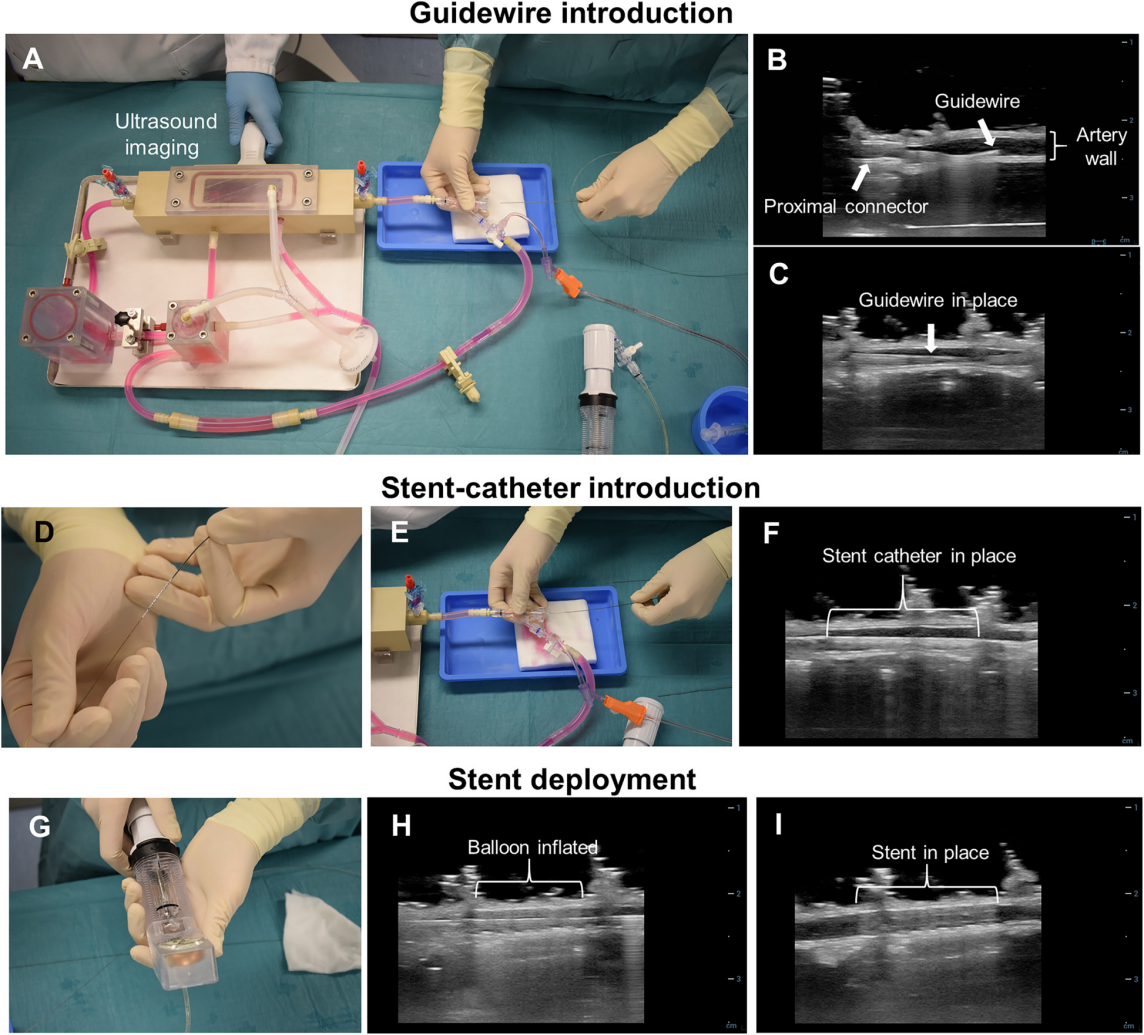
*Ex vivo* coronary stent implantation procedure. **(A)** *Ex vivo* stent implantation set-up. The implantation procedure is US-guided and the catheters are inserted in the direction of flow. **(B)** US image of the guidewire introduction from the proximal connector. **(C)** US image of the guidewire in place. **(D)** The guidewire is inserted in the stent delivery system and together **(E)** introduced in the vascular bioreactor through the rotating hemostatic valve. **(F)** US image of the stent catheter inside the artery. **(G)** A manual pump with pressure gauge (inflator) was used to inflate and deflate the balloon of the stent delivery system. **(H)** US image of the inflated balloon. **(I)** US image of the stent deployed into the artery.

The *ex vivo* stent implantation procedure is presented in Figure 3. To allow intravascular access during culture, the circuit of the bioreactor incorporated a rotating hemostatic valve (co-pilot, Abbott) proximal to the culture chamber (Figure 3A). Right before stent implantation, the *ex vivo* arterial diameter was measured in the region to stent using US imaging. The pump was stopped, and stent implantation was performed in a sterile manner. In order to maintain the pressure in the artery, the first resistance after the culture chamber (R2) was completely closed and a pressurized bag containing culture medium was connected to the circuit. The resistance between the pressurized media and the semi-closed circuit was adjusted manually to achieve and maintain the desired vessel diameter, as assessed by US. Stent implantation was performed using a standard over-the-wire technique as employed in clinical practice but guided by US instead of fluoroscopy. First, a guidewire was advanced through the circuit and the artery until it reached the R2 resistance (Figures 3B,C). Then, the balloon catheter with the stent was advanced, over the wire, until reaching the targeted artery segment (Figures 3D–F). The inflation pressure of the stent balloon was determined based on the *ex vivo* artery diameter and manufacturers' specifications to achieve a 10% overstretch of the artery lumen using 60 s of inflation (Figures 3G,H) as generally used in preclinical studies to avoid excessive damage. Bare metal stents (BMS) (Amazonia Croco, Minvasys, France) with a length of 16 mm and a nominal diameter of 2.75 mm (*n* = 2), 3 mm (*n* = 1) and 4 mm (*n* = 2) were implanted in the arteries from the in-house swine. Similar BMS (Kaneka, Japan) with a length of 13 mm and a nominal diameter of 3 mm (*n* = 1) and 3.5 mm (*n* = 1) were implanted in arteries dissected from slaughterhouse hearts. An US image showing the stent in place is presented in Figure 3I. After stent implantation, the bioreactor was re-connected to the pump and flow was re-started. Then, approximately 200 ml of medium was refreshed.

### 2.10 Culture protocol with the addition of an external support

**Figure 4:**
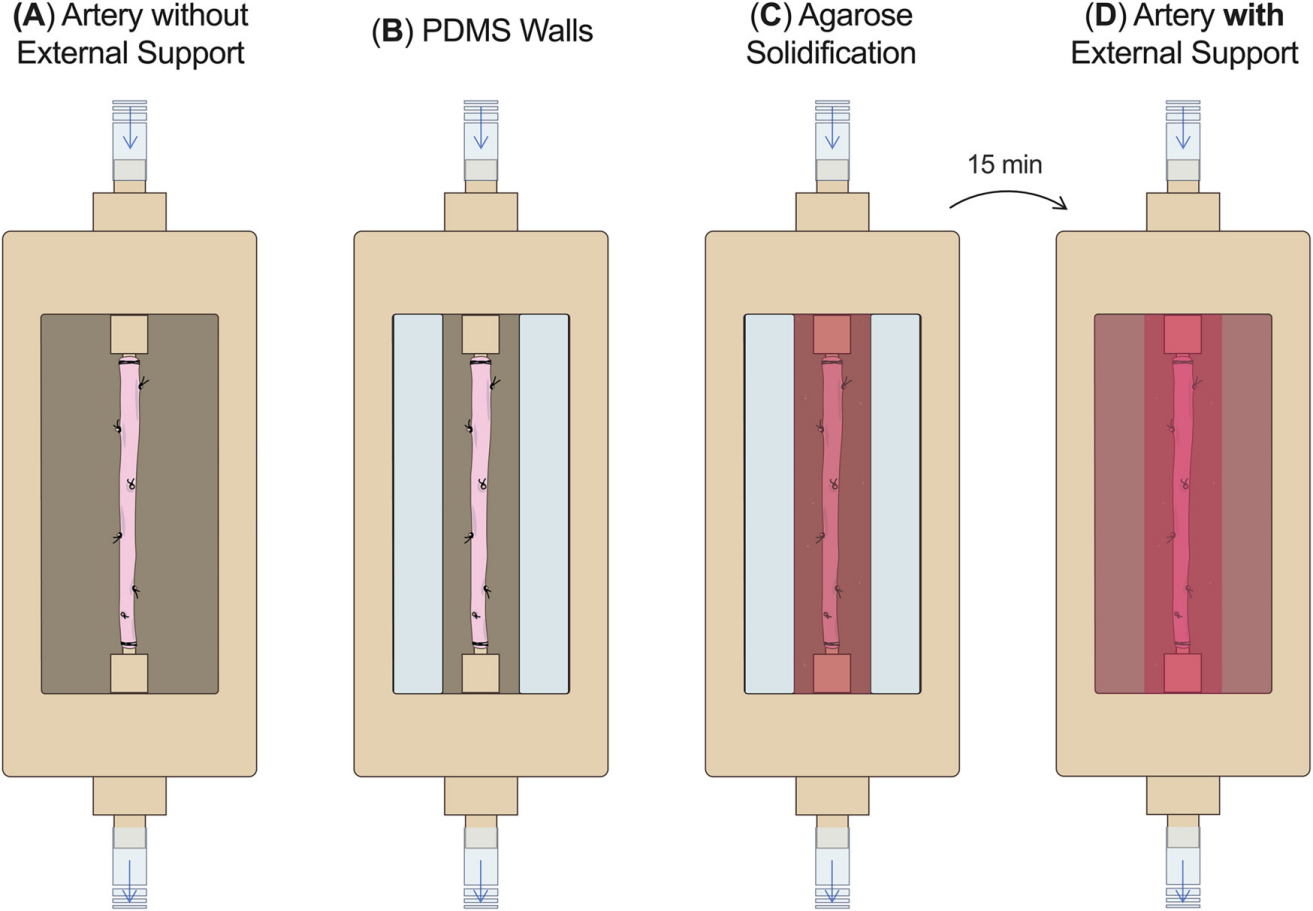
External support fabrication. **(A)** The RCA without the external support was mounted in the culture chamber without culture medium. **(B)** PDMS walls were positioned around the RCA. **(C)** The agarose solution was poured around the artery and **(D)** after 15 min the external support was solidified and culture medium was added around the artery. PDMS, polydimethylsiloxane.

To allow adding an external support around the artery right before the start of the culture, 2% agarose (Agarose, A9539-500G, Sigma-Aldrich, USA) was dissolved in DMEM, autoclaved, and was added to the compartmentalized bioreactor right before starting the culture (Figure 4). First, the artery without the external support was mounted in the autoclaved (1 h 121°C) and empty culture chamber and pressurized (Figure 4A). Then two walls of polydimethylsiloxane (PDMS) were positioned longitudinally to the artery to create a mold around it (Figure 4B). The cooled but liquid agarose solution (∼38°C, swine body temperature) was poured around the artery (Figure 4C). After 15 min, the agarose structure (10 × 2 × 3 cm) was solidified and the PDMS walls were removed (Figure 4D).

### 2.11 Histology

At the end of the cultures, the structural morphology of the RCAs was assessed in histologic preparations. After fixation, the RCAs proximal and distal from the stent were cut into 5 mm blocks, processed for paraffin embedding and sectioned (5 µm). The sections were stained for Hematoxylin and Eosin (H&E) and Resorcin Fuchsin (RF). The mid-stent segment was embedded in methyl methacrylate (MMA), cut into blocks, and stained en-face with toluidine blue (1%)-borax(2%) in water for 25 min at 45°C to visualize all stent strut positions.

The histologic evaluation was performed by an experienced pathologist (HvB). The morphology of the RCAs was considered maintained if the internal and external elastic lamina were intact, and no alternations were observed in the medial layer. For the stented segments, the presence of dissections was defined as any discontinuity between the intima, media or adventitia.

### 2.12 Data analysis and representation

All data are presented as mean ± standard deviation (SD). All graphs were generated by using GraphPad Prism (version 8.0.1). Statistical analysis was performed using IBM SPSS Statistics [version 28.0.1.0 (142)]. Changes in diameter between day 0 and day 3 and between day 3 and *in vivo* were studied using a paired T-test. Comparisons were made by pooling data from arteries without (Step 1A and 1B; *n* = 5) and with external support (Step 1C and 1D; *n* = 5).

## 3 Results

### 3.1 Final study design with iterations taken in step 1

The main goals of the study were to determine the time point for *ex vivo* stent implantation, the circumstances to perform *ex vivo* in-culture RCA stent implantation, and to use the gained insights to culture and stent RCAs from slaughterhouse tissue. The pragmatic step-by-step approach used to achieve this goal is outlined in Figure 1. For the in-house swine for which the *in vivo* diameter was known (*n* = 19), we gained insights on the following four situations: cultures without and with external support (ES−/ES+) and without and with bare metal stent implantation (BMS−/BMS+). Insights from the simplest iteration (Step 1A = ES-/BMS-) were translated to the next (Step 1B = ES−/BMS+), and so on, with the insights from Step 1D (ES+/BMS+) translated to the proof of principle of culturing and stenting of slaughterhouse swine in step 2 (*n* = 2). In Step 1A, we started determining the best time point to implant the stent during the *ex vivo* culture by measuring how long it takes for the RCA to stabilize to the *in vivo* diameter and remain stable for at least 24 h. In Step B (ES-/BMS+), stents were implanted in the RCAs after 4 days of *ex vivo* culture but this resulted in dissections within and at the edges of the stents. In order to reduce the risk of RCA dissections and to provide support to the vessel, an external support for the RCAs was created for Step 1C (ES+/BMS−). RCAs were cultured with the addition of the external support, resembling *in vivo* coronary artery surrounding arterial tissue. In these RCAs, stents were not yet implanted, in order to first check morphology and hemodynamics of the vessel with the addition of the external support. The RCAs were cultured longer to investigate if the morphology of the artery was maintained, which was confirmed by histology. In Step D (ES+/BMS+), RCAs were cultured with the external support and stents were implanted at day 3 of culture (based on the outcome of the previous step) and maintained for 2 additional days, similar to preclinical studies (10). Once optimization of stent implantation in the *ex vivo* vascular bioreactor was completed, in Step 2 (ES+/BMS + in slaughterhouse swine coronary arteries), RCAs were dissected to show the potential of this type of waste material. Stents were implanted after 3 days of culture, and the RCAs could be maintained in culture for additional 2 (*n* = 1) and 5 days (*n* = 1 as a proof of principle, with timepoints similar to one of our preclinical studies (10).

### 3.2 Outcome step 1A—the earliest time point for in-culture stent implantation is after 3 days of culture

**Figure 5:**
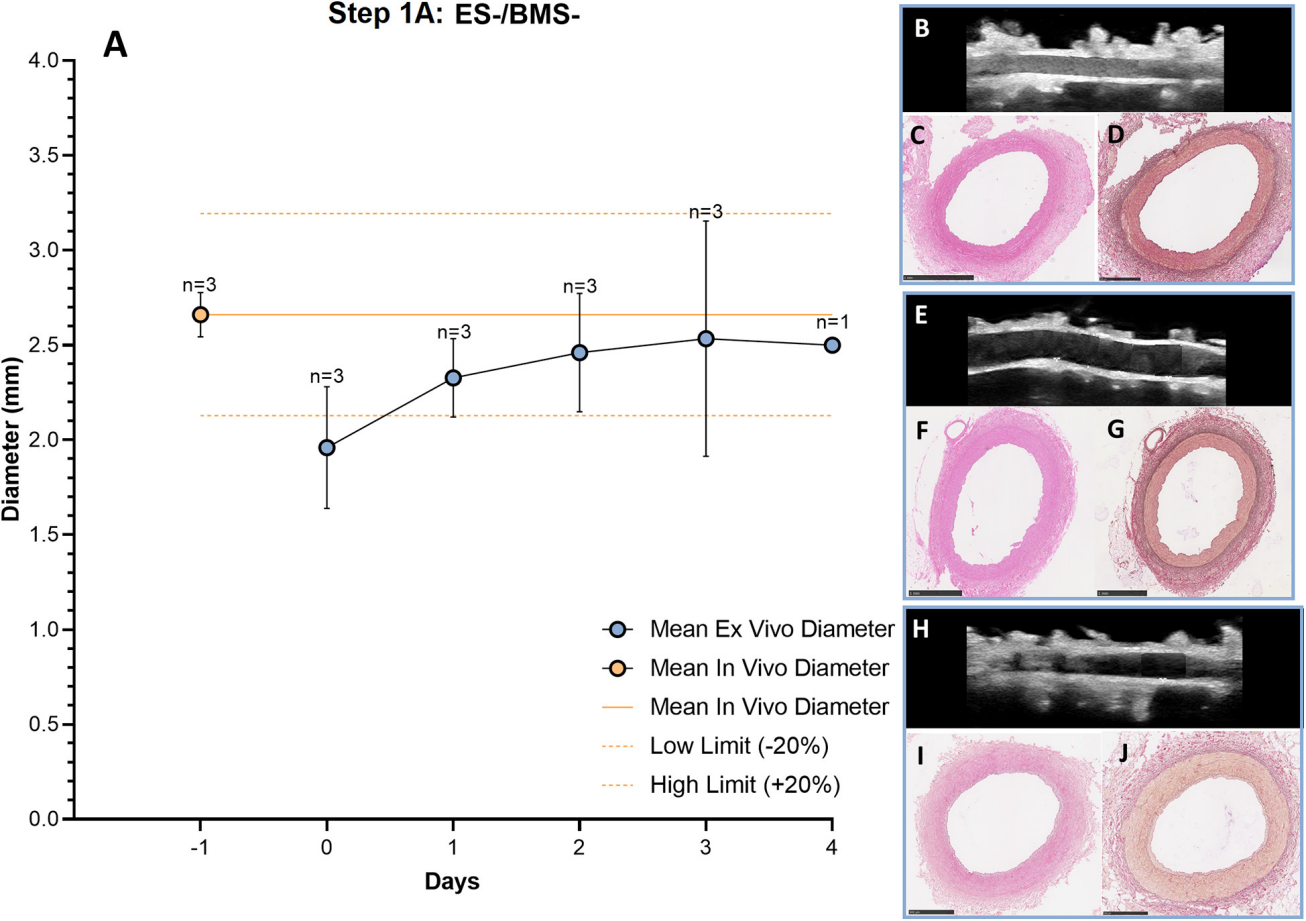
Outcome step 1A—*Ex vivo* culture of RCAs from in-house swine (ES-/BMS-). **(A)** The mean *ex vivo* diameter increased in the first 3 days and it reached stability between day 3 and day 4 (graph). The solid orange line indicates the value of the *in vivo* diameter as measured by angiography. The dashed orange lines indicate the *in vivo* diameter range (+/− 20%). Data are presented as Mean ± SD. **(B–J)** US **(B, E, H)** of the RCAs and histology of their central portion (H&E staining in C, F and I and RF staining in D, G and J) for RCAs cultured for 3 days [*n* = 2, **(B–D)** and **(E–G)**] and 4 days [*n* = 1, **(H–J)**]. US images were post-processed to digitally remove the engrafted measurements. ES− = without external support; BMS− = without stent. Scale bar: 1 mm.

The RCAs were cultured in the *ex vivo* vascular bioreactor for 3 (*n* = 2) and 4 (*n* = 1) days. The *in vivo* diameter (+/- 20%) was reached after 3 days of culture and the *ex vivo* diameter in the bioreactor was considered stable between day 3 and day 4 (Figure 5A). During culture, the structure of the vessel was maintained intact, as assessed by US imaging performed daily. In Figures 5B–H, US images of the central portion of the 3 different *ex vivo* cultured RCAs are presented together with the corresponding histology (H&E staining in Figures 5C–I and RF staining in Figures 5D–J). The histology showed RCAs that appeared normal with an intimal layer bounded by the internal elastic lamina and a cell-rich media layer bounded by an external elastic lamina and adventitia. The average peak endothelial shear stress during the length of the cultures was 2.0 ± 1.1 Pa and the corresponding average peak flow was 45 ± 11 ml/min. The average MAP was 18 ± 11 mmHg. The complete list of the hemodynamic parameters of the *ex vivo* cultures from Step 1A to 1B calculated per day are presented in Supplementary Table S1, and *in vivo* values are given in Supplementary Table S2.

### 3.3 Outcome step 1B—in-culture stent implantation causes dissections in *ex vivo* cultured RCAs

**Figure 6:**
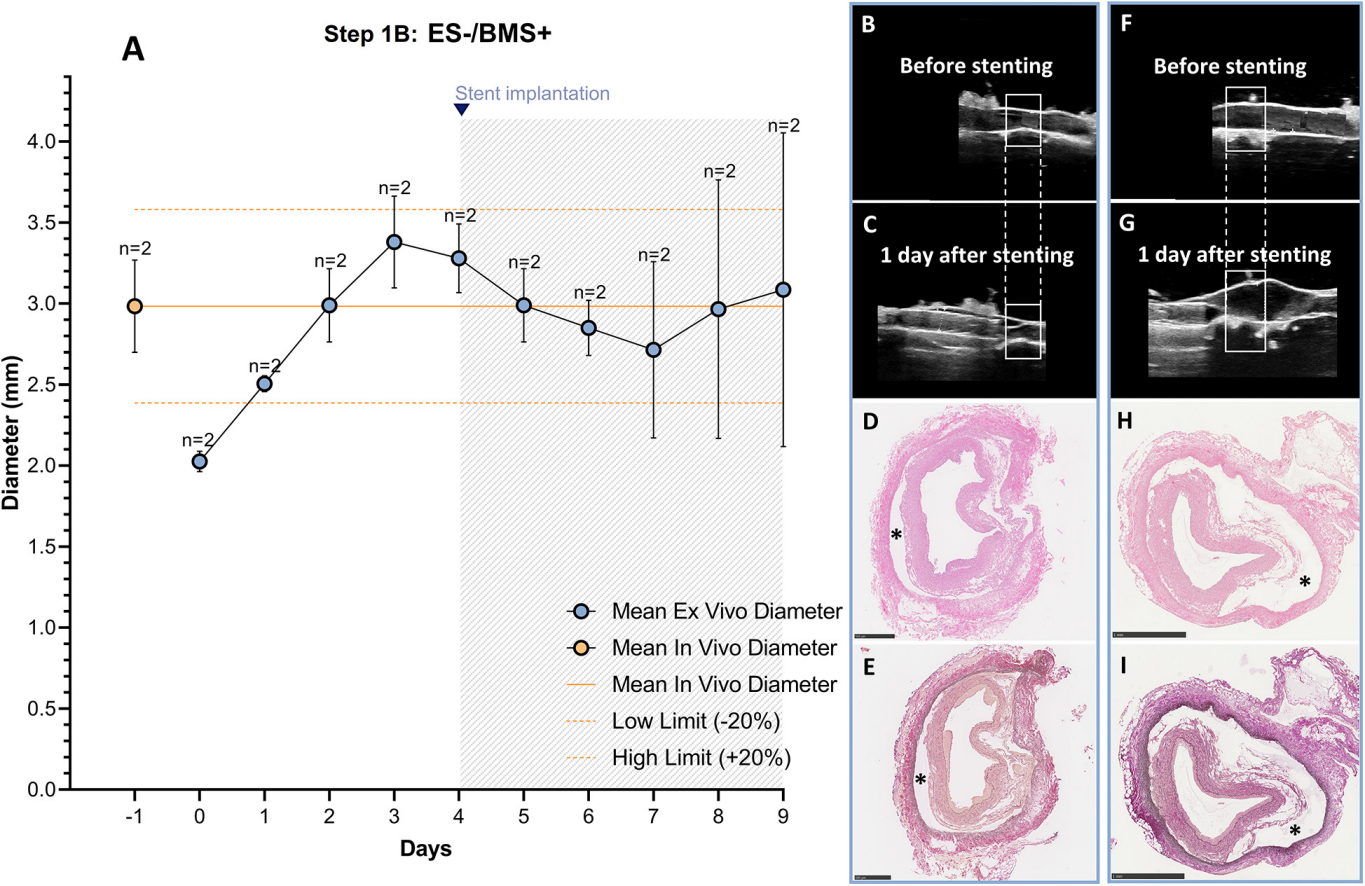
Outcome step 1B—*Ex vivo* culture of RCAs from in-house swine with stenting (ES-/BMS+). **(A)** The stents were implanted when the *ex vivo* diameter was stable and in the range of the *in vivo* diameter (day 4, graph). The solid orange line indicates the value of the *in vivo* diameter as measured by angiography. The dashed orange lines indicate the *in vivo* diameter range (+/− 20%). Data are presented as Mean ± SD. **(B–I)** Right panel images of US and corresponding histology from RCAs cultured for 5 days after stenting [*n* = 2, **(B–E)** and **(F–I)**]. **(B,F)** US images of the region before stenting the two different RCAs. **(C,G)** US images show the stented region 1 day after stenting for the two different RCAs. After stent implantation, a dissection in the vessel wall is visible. US images were post-processed to digitally remove the engrafted measurements. **(D,H)** H&E staining at the end of culture of the two different RCAs; **(E,I)** RF staining at the end of culture of the two different RCAs. (*) dissections between the media and the adventitia layers. ES− = without external support; BMS+ = with stent. Scale bar: 1 mm.

The RCAs were cultured in the *ex vivo* vascular bioreactor for 9 days (*n* = 2). The stents (*n* = 2) were implanted when the *ex vivo* diameter was stable and in the range of the *in vivo* diameter. This occurred at day 4 (Figure 6A). Before stent implantation, no dissections were seen with US imaging (Figures 6B,F). The day after stent implantation (day 5), dissections between the media and the adventitia were visible. The dissections were located within and at the edges of the stented region (Figures 6C,G). Histology was performed to confirm the dissections that are visible between the tunica media and adventitia, indicated by an asterisk in (Figures 6D–I). The average MAP during the first 4 days of culture was 30 ± 8 mmHg, the average peak flow was 37 ± 8 ml/min and the average peak endothelial shear stress was 1.0 ± 0.6 Pa. These values are the average of the hemodynamic parameters before the dissections were visible. After dissection, the hemodynamic parameters were considerably influenced, and we excluded the data from the reported values.

We used a paired T-test and found, for the in-house swine groups 1A and 1B, without the external support, a significant increase in diameter between day 0 and day 3 (two-sided *p* = 0.022) which resolved at day three with no significant difference between day 3 and *in vivo* (two-sided *p* = 0.6), showing that the diameters had indeed returned to the *in vivo* diameter (see Supplementary Figure S1 and Supplementary Table S2).

### 3.4 Outcome step 1C—the addition of external support to the RCAs allows for a better physiological tuning of the hemodynamics

**Figure 7:**
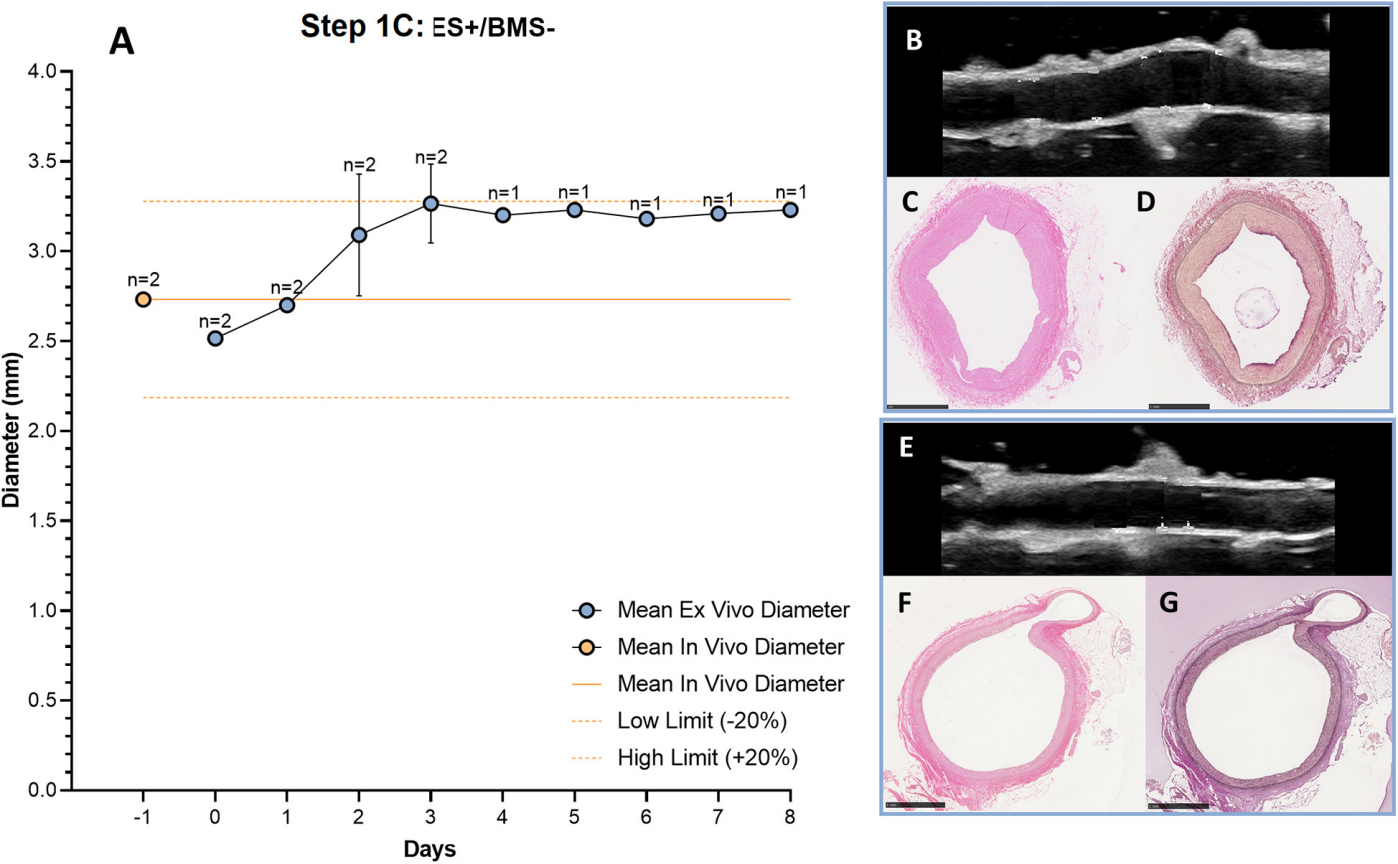
Outcome step 1C—*Ex vivo* culture of RCAs from in-house swine with the external support (ES+/BMS-). **(A)** The mean *ex vivo* diameter increased in the first 3 days and it reached stability between day 3 and day 4 (graph). The solid orange line indicates the value of the *in vivo* diameter as measured by angiography. The dashed orange lines indicate the *in vivo* diameter range (+/− 20%). Data are presented as Mean ± SD **(B–G)** US **(B,E)** of the RCAs and histology of their central portion (H&E staining in C, F and RF staining in D, G) for RCAs cultured for 3 days [*n* = 1, **(B–D)**] and 8 days [*n* = 1, **(E–G)**]. US images were post-processed to digitally remove the engrafted measurements. ES + = with external support; BMS− = without stent. Scale bar: 1 mm.

**Figure 8:**
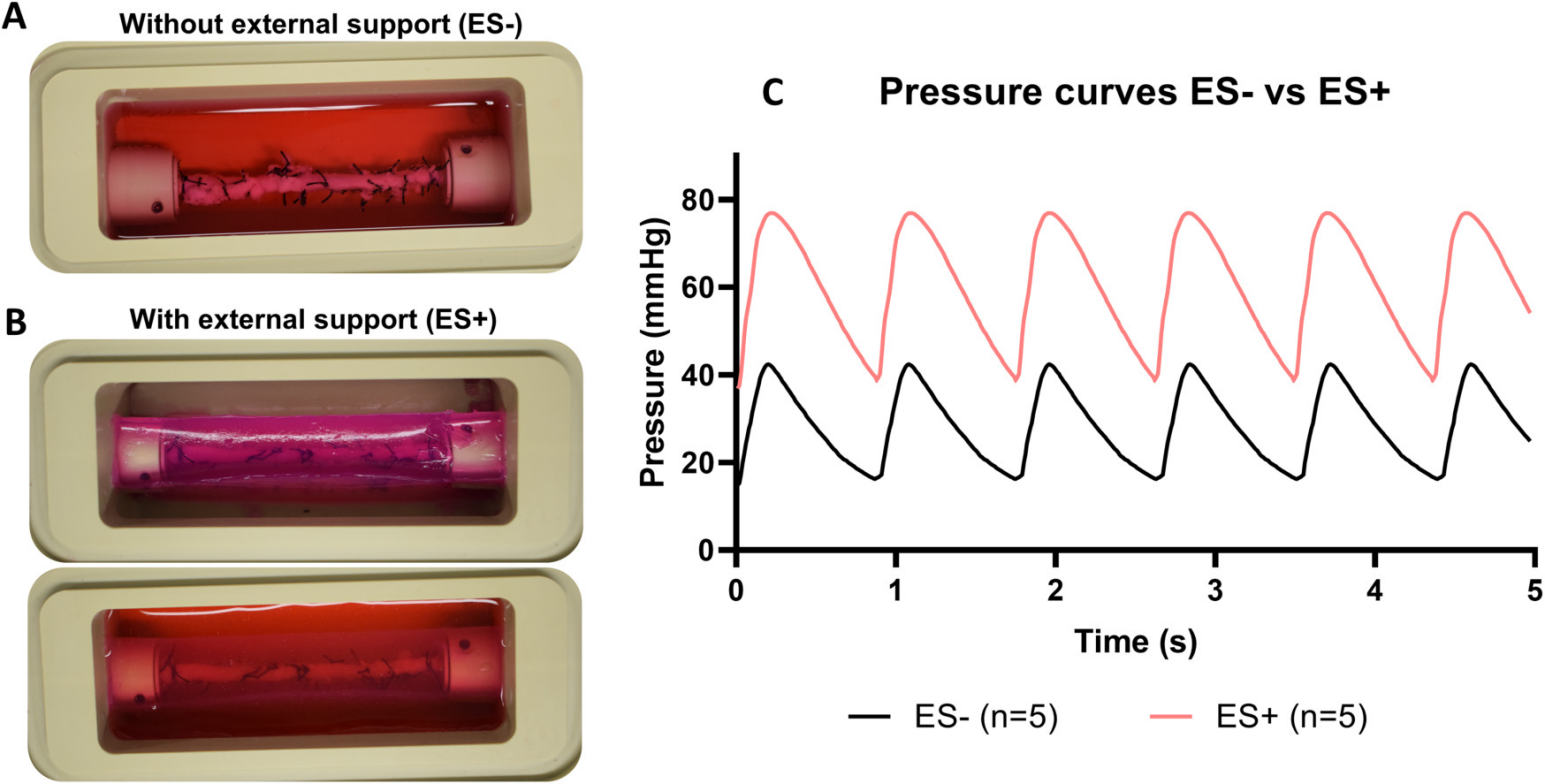
*Ex vivo* cultures before and after the introduction of the external support **(A)** RCA in the culture chamber surrounded by culture medium. **(B)** RCA in the culture chamber with the addition of the external support (above) and surrounded by culture medium (below). **(C)** Pressure curves comparison with (ES+) and without (ES−) the external support. Pressure curves are the average of pressure for the arteries at day 3 of culture from ES− (Step 1A and B) and ES+ (Step 1C and D). Black line = average of pressure curves generated by *ex vivo* culturing arteries without the external support. Red line = average of pressure curves generated by *ex vivo* culturing arteries with the external support. ES− = without external support; ES+ = with external support.

To optimize stent implantation and to avoid dissections of the vessel wall, an external support to the artery was created using agarose. RCAs were cultured *ex vivo* in the bioreactor for 3 (*n* = 1) and 8 days (*n* = 1) to assess the vessel morphology and structure with the introduction of the external support. Similar to the results without support, the diameter was stable and in the range of the *in vivo* diameter after 3 days of culture (Figure 7A). US imaging showed an intact structure of the 2 RCAs at the end of the culture (Figures 7B,E) and their morphology was maintained during the culture (day 3: Figures 7C,D; day 8: Figures 7F,G). While the RCAs were unsupported in the culture chamber in Steps 1A-B (Figure 8A), in step 1C the RCA received support from the agarose (Figure 8B). With this external support, the RCAs could be cultured with higher arterial pressures (Figure 8C). The average MAP during the length of the cultures was now 42 ± 7 mmHg, and considerably higher than in the cultures without support in Steps 1A–B. The average peak flow was 48 ± 11 ml/min and the corresponding average peak endothelial shear stress was 1.0 ± 0.4 Pa.

### 3.5 Outcome step 1D—the external support prevents dissections when stenting in-culture

**Figure 9:**
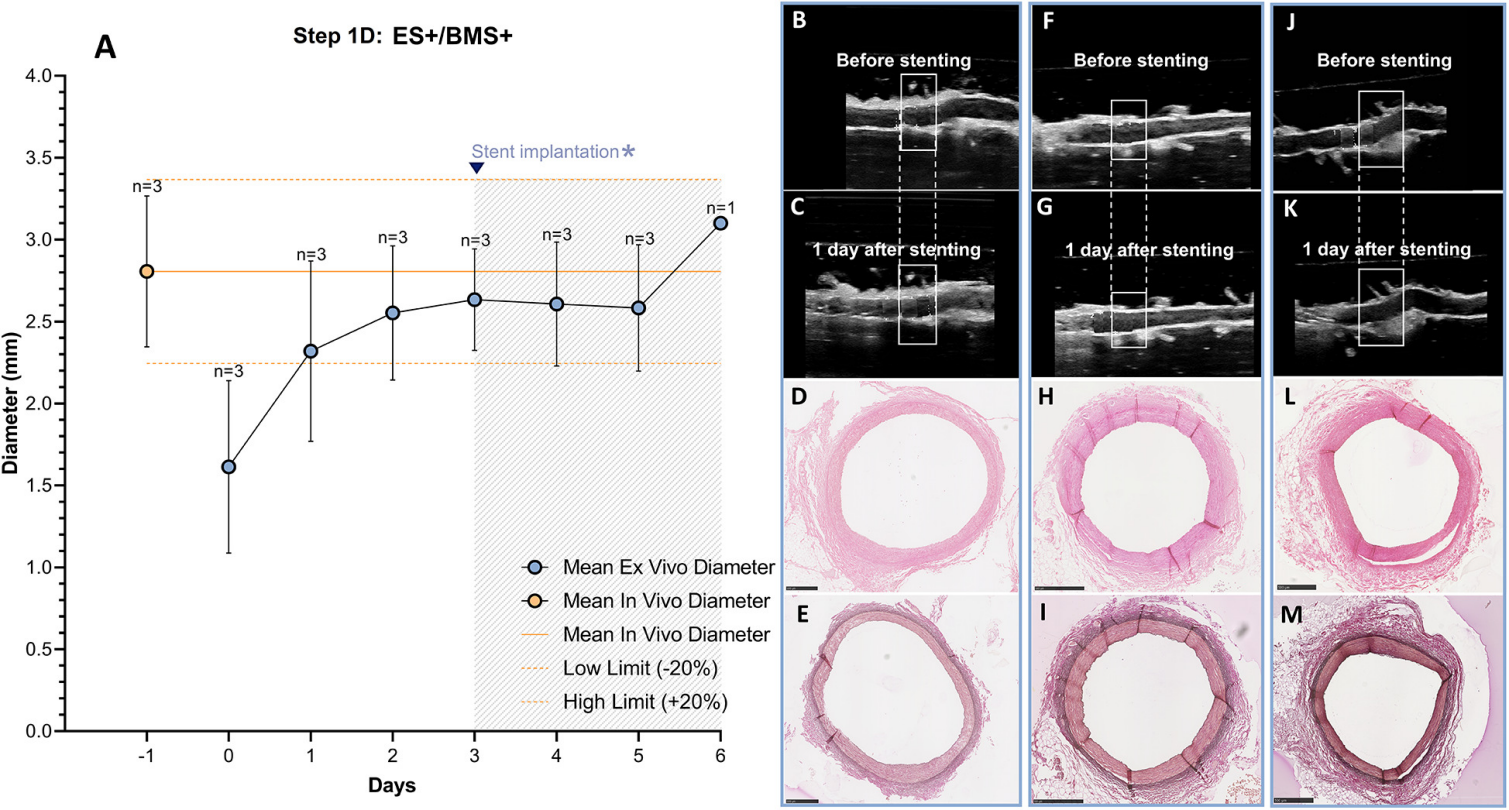
Outcome step 1D—*Ex vivo* culture of RCAs from in-house swine with the external support and with stenting (ES+/BMS+). **(A)** The stents were implanted when the *ex vivo* diameter was stable and in the range of the *in vivo* diameter (day 3, graph). The solid orange line indicates the value of the *in vivo* diameter as measured by angiography. The dashed orange lines indicate the *in vivo* diameter range (+/− 20%). Data are presented as Mean ± SD. **(B–M)** US (B/C, F/G and J/K) of the RCAs and histology of their central portion (H&E staining in D, H and L and RF staining in E, I and M) for RCAs cultured for 2 days after stenting [*n* = 2, **(B–E)** and **(F–I)**] and 3 days after stenting [*n* = 1, **(J–M)**]. US images were post-processed to digitally remove the engrafted measurements. ES− = without external support; BMS+ = with stent. Scale bar: 1 mm.

**Figure 10:**
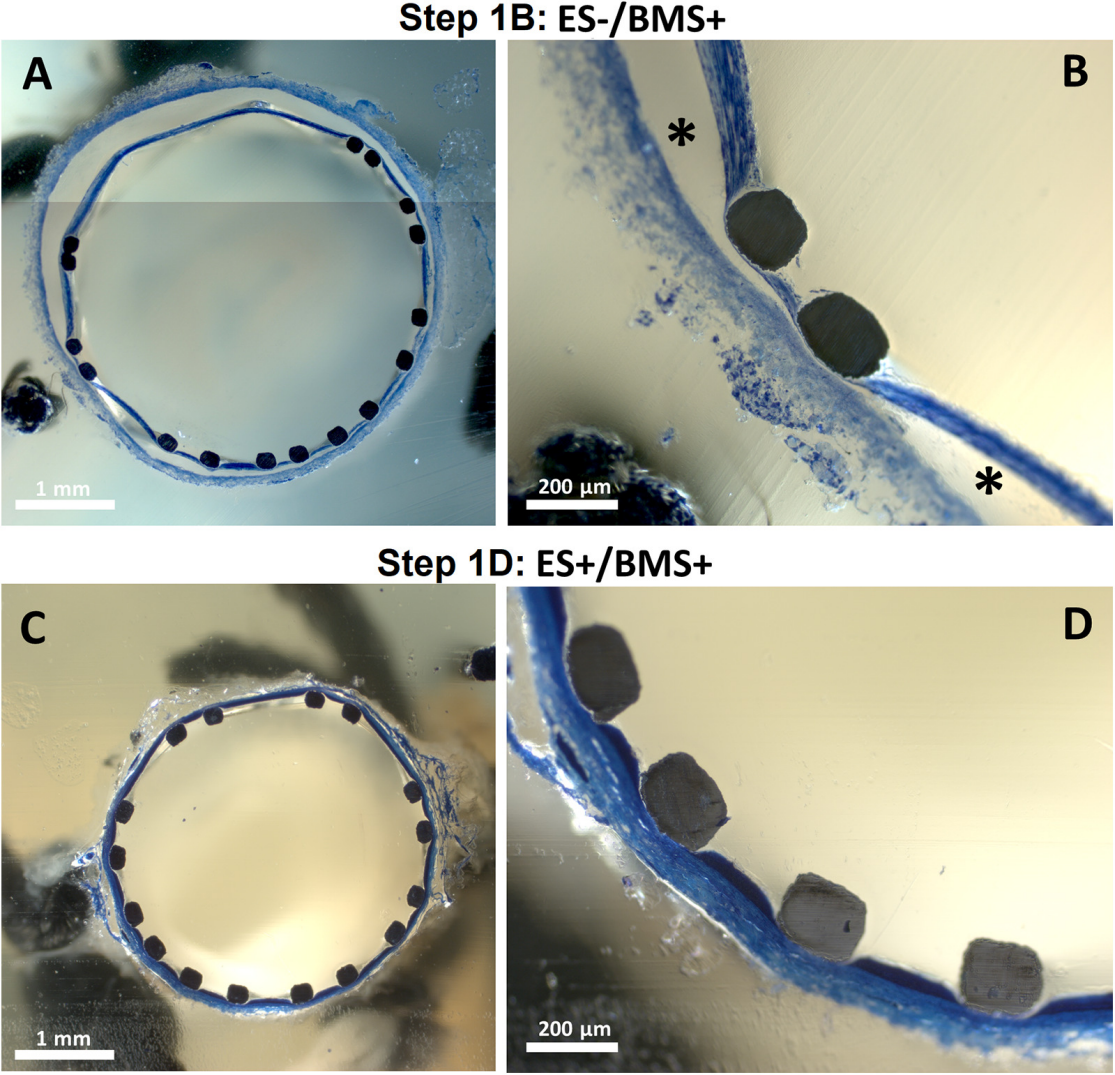
Toluidine blue staining of stented segments. One representative artery is chosen for steps where stents were implanted in RCAs from in-house swine (Step 1B and D) **(A,B)** Central portion of the stented segment for one RCA cultured in Step 1. * indicates dissected areas between the tunica media and adventitia. **(C,D)** Central portion of the stented segment for one RCA cultured in Step 1D.

The RCAs (*n* = 3) with the external support were cultured in the *ex vivo* vascular bioreactor until the *ex vivo* diameter was stable (3–4 days) and in the range of the *in vivo* diameter (Figure 9A). Stents were therefor implanted at day 3 (*n* = 2) and at day 4 (*n* = 1). The US images showed an unaltered morphology of the distal part to the stent before (Figures 9B–J) and one day after stent implantation (Figures 9C–K). Moreover, according to histology, the morphology at microscopic level was also maintained with no signs of damage (H&E staining in Figures 9D–L and RF staining in Figures 9E–M). Further analysis of the stented regions through toluidine blue staining showed that dissections that were clearly visible between the media and the adventitia in Step 1B (Figures 10A,B) were prevented now by the introduction of the external support (Figures 10C,D). Moreover, compared to the arteries of group ES-/BMS + (Step 1B), the pressure could be maintained at higher levels as for the arteries of group ES+/BMS- (Step 1C). The average MAP during the entire length of the cultures was 59 ± 7 mmHg. The average peak flow was 39 ± 9 ml/min and the corresponding average peak endothelial shear stress was 1.4 ± 0.8 Pa.

We used a paired *T*-test and found, for the in-house swine groups 1C and 1D with the external support, a significant increase in diameter between day 0 and day 3 (two-sided *p* = 0.02) which resolved at day three with no significant difference between day 3 and *in vivo* (two-sided *p* = 0.8), showing that the diameters had indeed returned to the *in vivo* diameter (see Supplementary Figure S1 and Supplementary Table S2).

### 3.6 Outcome step 2—in-culture stent implantation can be replicated in slaughterhouse RCAs with external support

**Figure 11:**
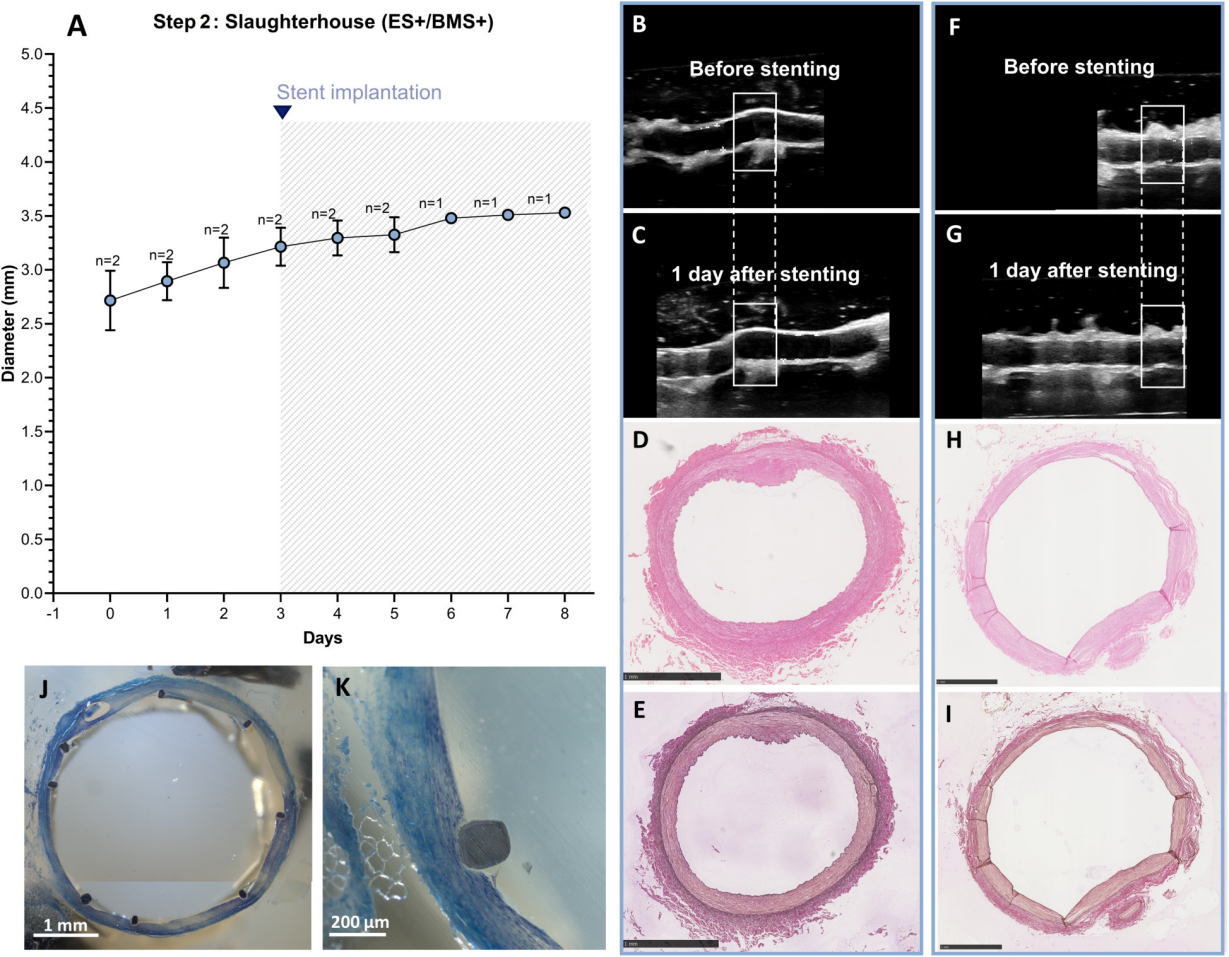
Outcome step 2—*Ex vivo* culture of RCAs from slaughterhouse swine with the external support and with stenting. **(A)** The stents were implanted at day 3 of culture (graph). Data are presented as Mean ± SD. **(B–I)** US (B/C, F/G) of the RCAs and histology of their central portion (H&E staining in D, H and RF staining in E, I) for RCAs cultured for 2 days after stenting [*n* = 1, **(B–E)**] and 5 days after stenting [*n* = 1, **(F–I)**]. US images were post-processed to digitally remove the engrafted measurements. **(J,K)** Toluidine blue staining of the stented segment for the RCA cultured for 5 days after stenting showing the presence of nuclei stained dark blue. ES+ = with external support; BMS+ = with stent. Scale bar: 1 mm.

After optimization of the *ex vivo* coronary stent implantation approach in Steps 1A–D, using arteries with known *in vivo* diameter from in-house swine, RCAs isolated from swine hearts harvested from the slaughterhouse with unknown diameters were used for the experiments. RCAs were cultured in the *ex vivo* vascular bioreactor until the diameters were stable at day 3 (Figure 11A), and stents (*n* = 2) were implanted. The stented RCAs were maintained in culture for an additional 2 days (*n* = 1) and 5 days (*n* = 1) as a proof of principle, similar to a previous preclinical study (10). The US images showed the RCA segment distal to the stent before (Figures 11B,F) and the day after stent implantation (Figures 11C,G). The structure of the vessel wall was intact, with no visible dissections. The morphology was maintained with no signs of damage (Figures 11D–I). Also, through toluidine blue staining, no signs of damage were observed (Figures 11J,K). The average MAP during the length of the cultures was 63 ± 7 mmHg. The average peak flow was 61 ± 15 ml/min and the corresponding average peak endothelial shear stress was 1.0 ± 0.2 Pa.

## 4 Discussion

To the best of our knowledge, this is the first study performing in-culture coronary stenting, guided by US imaging, using an *ex vivo* vascular bioreactor. The main results of our pragmatic iterative approach to optimize in-culture stent implantation of RCAs in the *ex vivo* VABIO bioreactor are listed below and are discussed in detail in the following subsections:

• RCAs needed time to adapt to the *ex vivo* culture. In terms of diameter stability (based on *in vivo* measurements), the earliest time point to perform in-culture stent implantation was after 3 days of *ex vivo* culture;
• Despite the stable diameter of the RCAs, in-culture stenting led to mural dissections. The introduction of the RCA external support in the form of an agarose matrix surrounding the artery prevented dissections during stenting and also allowed for higher intravascular pressures during culture;
• The protocol for in-culture stenting of RCAs optimized for in-house swine (with a known *in vivo* diameter) successfully worked for slaughterhouse RCAs (with an unknown *in vivo* diameter).

### 4.1 Coronary arteries need to adapt to *ex vivo* culture conditions before stenting

In pre-clinical and clinical stenting of any vascular bed, the relation between stent and artery diameter has a significant influence on the results of the procedure. For example, large overstretching of the artery or malapposition of the stent struts (under-deployment) are both related to poor outcome (12). In *ex vivo* settings, understanding the possible variations and adaptation in the first days of culture compared to the *in vivo* diameter would represent a substantial advantage for stent/balloon selection. It helps to better mimic *in vivo* conditions to better predict outcomes. In our study, we had the opportunity to measure the *in vivo* RCA diameter with angiography prior to *ex vivo* culture. At the beginning of the culture, we observed a reduction of RCAs diameter of approximately 30% compared to the *in vivo* diameter. However, during the first days of culture, all arteries adapted to the new environment with an increase in diameter, which stabilized to the *in vivo* diameter range after 3 days. Our observations regarding the changes in diameter are in agreement with previous studies using carotid arteries in the VABIO platform, which also showed that the *ex vivo* diameter gradually increased during the first 3 days of culture (6). This adaptation behavior has been attributed to changes in pressure and temperature experienced by the arteries in the first days of culture (6, 13, 14). In our experiments, we mostly attributed the initial RCA contraction to the cooling of the tissue to 4°C prior to *ex vivo* culturing. However, cooling is needed to preserve the viability of the tissue during transport and handling in most of the *ex vivo* settings. Moreover, other factors such as stress during sacrifice (for slaughterhouse animals) or direct manipulation of the vessel wall during dissection could also play a role. Despite the initial RCA contraction that may occur in *ex vivo* cultures due to the above-mentioned factors, it is noteworthy that the vessel can recover its *in vivo* diameter during culture as we demonstrated in our study. Understanding the dynamics of the artery when adapting to normothermic (38°C, for swine) and pressurized culture is key when the diameter of the artery is as relevant as it is in stent implantation.

### 4.2 Optimization of *ex vivo* stent implantation procedure: introduction of the external support

As already mentioned, the main goal of our study was to perform in-culture stenting in RCAs. Once we defined a time window where the artery diameter was stable in culture, we stented the RCAs in a similar manner to that used in patients. However, our stent implantation procedures damaged the arterial wall causing mural dissections, which were visible with US imaging starting from the day after stent implantation (Figure 6), a phenomenon that is not usually seen in *in vivo* stent implantation. This could be ascribed to different factors such as overall deterioration of the artery by the culture process or the occurrence of acute force changes in specific positions during stent implantation, which can create weak points. However, according to histology, the general morphology of the artery was well maintained at the moment of stent implantation (i.e., after day 3, Figure 5). Therefore, we hypothesized that the primary cause of the dissections during stent implantation was the absence of an external tissue supporting the artery when it overstretches and collapses during stenting. *In vivo*, perivascular tissue has many functions, among which to maintain vascular structure and to regulate vascular functions (15). In absence of supporting perivascular tissue, as reported in Step 1 of our study, the vessel wall alone cannot prevent the complete collapse of the artery when pressure drastically drops (e.g., for both stopping and restarting the pump for stenting) or limit the overstretching during stent deployment. Aiming to provide external support to our vessels in culture, we added an artificial external support made of agarose. This polysaccharide is an excellent matrix for cell and tissue cultures due to its chemical and mechanical properties such as the controlled self-gelling property which facilitates the molding of agarose in specific shapes or, as in our case, around complex structures like vessels. Moreover, agarose has excellent biocompatibility, crucial for long culture periods, and low costs (16). Agarose has been used as an external support matrix for *ex vivo* stenting in a previous study, but it was only tested in acute experiments using frozen arteries (17). Here, we showed that the agarose matrix can be used in alive arteries undergoing long-lasting culture and that it prevented vessel wall dissections during in-culture stenting.

The addition of an artificial external support was not only essential for the stenting procedure, but it also improved the tuning of intravascular hemodynamics, allowing to reach higher pressures. In our experiments, the addition of the external support allowed us to double the intravascular pressure, reaching up to 57 ± 5 mmHg of MAP (Step 1D, ES+/BMS+). The pressures that we achieved in our study are still lower compared to physiological conditions (70–100 mmHg, MAP), but they are significantly higher than the pressures previously reported for coronary arteries. To the best of our knowledge, only two studies performed coronary stent implantation using an *ex vivo* vascular bioreactor (5, 9), with only Wang et al. reporting hemodynamic parameters (5). In their study, the coronary arteries were cultured under a pressure range of 40–5 mmHg (SAP-DAP), a peak flow of 18.3 ml/min and a peak wall shear stress of 0.68 Pa. In our study, the RCAs were cultured with a pressure range of 45–15 mmHg before the introduction of the external support, resembling the results given by Wang et al. Of note, in our study, the pressure range increased up to approximately 80–35 mmHg (SAP-DAP) with the introduction of the external support. Pressures and shear stress were also consistently higher when using the optimized protocol of Step 1D in slaughterhouse arteries, as discussed in the following section.

Further studies are needed to investigate the relevance of tuning pressure at physiological conditions and how to achieve that. For example, by modifying the stiffness of the external support matrix using different concentrations of agarose or adding or using other compounds. However, when designing novel external support matrices, a balance between the provision of support to the vessel and capability of the matrix to stretch should be considered. In our setting, this balance was achieved with 2% agarose, which allowed pulsatility and distension of the artery during stent implantation while giving enough support to significantly increase pressure and preventing dissections.

### 4.3 Culture and stent implantation in slaughterhouse arteries

So far, in most of the studies using *ex vivo* bioreactors the stent was implanted before starting the culture (5, 9, 18–20), and the reporting of the desired and achieved stent-to-artery ratio, as well as changes in diameter during culture, is limited. In Step 2, we successfully implemented our protocol on how to tune the *ex vivo* hemodynamic parameters, based on the in-house swine (known *in vivo* RCA diameter), in slaughterhouse RCAs. Moreover, we used US imaging for in-culture stent implantation with a stent-to-artery ratio of 1.1:1, an overstretch commonly used in pre-clinical studies (10). As a result of the optimized protocol (Step 1D), we could stent during culture without complications (mural dissections) and better resemble the pre-clinical procedures.

In terms of culture settings, when applying the optimized protocol in slaughterhouse arteries, remarkably, this resulted in even higher pressures (63 ± 3 mmHg, MAP) compared to the cultured RCAs of in-house swine, being closer to, but not yet at, physiological conditions. This could be explained because the slaughterhouse RCAs had a bigger diameter compared to in-house swine (2.7 ± 0.3 mm vs. 2.0 ± 0.4 mm at the start of culture, respectively). In our experiments, hemodynamics were tuned to maintain an *in vivo* wall shear stress (0.6–2.1 Pa) (11, 18) and, in our system, bigger arteries required higher flow and a subsequent higher pressure to maintain a endothelial shear stress of 1.30 ± 1.0 Pa. The differences in diameter of the RCAs could be explained by the different body weight of the animals. Although the information of the exact body weight (and age) of the slaughterhouse animals was not available for us, pigs are normally sacrificed with a body weight around 100 kg, compared to the 40–50 kg of the in-house animals. The variations in the diameter of the arteries between different animal sources might be an important factor to consider when setting up *ex vivo* studies and ascertaining they fit in the bioreactor, since it can significantly influence the possibilities of hemodynamic tuning depending on the bioreactor system, as in our case. However, diameters reached were clinically relevant.

Another important aspect to mention when setting up *ex vivo* coronary studies based on previous studies is that most of the available literature is based on carotid arteries (8, 18–24) while only a few studies investigated *ex vivo* stent implantation in coronary arteries (5, 9). Of note, coronary arteries present some challenges when culturing them under pressure compared to carotid arteries, due to their differences (25). Coronary arteries especially present a significant number of side branches that need to be closed prior to culturing to maintain the pressure. Despite the challenges related to culture coronary arteries, we showed that, after optimization of the protocol, stent implantation can be performed in slaughterhouse RCAs**.** Our protocol could be already used to do a first screening of new catheter-based vascular interventions or intravascular imaging systems before the *in vivo* validation.

Both drug eluting balloons and drug eluting stents could theoretically also be studied in these bioreactors. It is important to realize that in the body, clearance reduces circulating drug levels within hours to negligible levels, depending on the chosen drug and its release pattern. Hence, the systemic levels should be modeled in the bioreactor by titrating medium refreshment during these early timepoints.

### 4.4 Limitations

Our study has several limitations. First, we included a relatively small number of arteries in the study and, some of them, were excluded from the final analysis, mainly due to infection. We only had access to a limited number of in-house swine where we could perform an *in vivo* coronary angiography, but we were able to iterate to improve relevant conditions. Regarding the number of slaughterhouse arteries, we were limited by the reduced stock of large stents (>3 mm). Second, the BMS used for in-house and slaughterhouse RCAs are different, but the Amazonia Croco model was not available for us at the moment of the experiments of Step 2. Nevertheless, we did not expect big differences regarding stent deployment between these two models. We did not have information on sex of the slaughterhouse swine. In case sex is important, small-scale slaughterhouses can supply such information. In addition, we were unable to mimic the early thrombotic and inflammatory response. Next steps should definitely include these aspects that add a layer of complexity to the bioreactor but bringing it closer to the *in vivo* situation. Finally, the VABIO was unable to mimic the three dimensional aspects of the heart and also contractility was not accounted for. This may change the response in the VABIO for longer-term studies.

## 5 Conclusion

Here, we showed the importance of time, hemodynamic tuning based on physiological shear stress, and the implementation of an external support in the stabilization of the diameter of coronary arteries when doing in-culture stenting of in an *ex vivo* vascular bioreactor. Moreover, our pragmatic two-step study demonstrated that in-culture US-guided stent implantation was feasible and optimized by adding external support to the artery. The resulting methodology paves the way for the use of slaughterhouse arteries in *ex vivo* coronary stenting experiments.

## Data Availability

The raw data supporting the conclusions of this article will be made available by the authors, without undue reservation.
