# Supplementary material for: In-culture coronary stenting in an ex vivo vascular bioreactor
Source: Front Cardiovasc Med. 2025 Jul 31;12:1565674. doi: 10.3389/fcvm.2025.1565674 (PMC12350248; doi:10.3389/fcvm.2025.1565674)
Supplement: Supplementary file 1 [file Datasheet1.pdf]

frontiers

SUPPLEMENTARY MATERIAL

In-culture coronary stenting in an *ex vivo* vascular bioreactor

F. Razzi[1§], J. Bobi[1§], M. Stijnen[2], J.H. van Esch[3], D.J. Duncker[1], V. van Steijn[3#], H.M.M. van Beusekom[1#*]

*** Correspondence:**

Heleen M.M. van Beusekom, PhD, Division of Experimental Cardiology, Department of Cardiology, Erasmus MC, University Medical Center Rotterdam, Office Ee2393a, Wytemaweg 80, Rotterdam 3015CN, Netherlands. E-mail: h.vanbeusekom@erasmusmc.nl

Supplementary Table 1

	Time-point (days)	D (mm)	SP (mmHg)	DP (mmHg)	MAP (mmHg)	Qpeak (ml/min)	SSpeak (Pa)	n
**ES-/BMS-**	0	2.0±0.3	33±18	6±4	15±9	40±14	3.0±1.8	3
	1	2.3±0.2	33±22	7±6	15±11	45±3	1.9±0.6	3
	2	2.5±0.3	40±27	7±6	18±13	47±18	1.6±0.3	3
	3	2.5±0.6	37±29	8±6	18±13	49±12	1.8±1.1	3
	4	2.5	70	15	33	41	1.3	1
**ES-/BMS+**	0	2.0±0.1	38±18	19±15	25±16	33±9	2.0±0.4	2
	1	2.5±0.1	50±21	22±14	31±16	34±11	1.1±0.4	2
	2	3.0±0.2	47±12	23±7	31±9	37±6	0.7±0.3	2
	3	3.4±0.3	49±5	22±1	31±1	44±16	0.6±0.4	2
	4	3.3±0.3	43±3	24±2	30±0	38±2	0.6±0.1	2
	5	3.0±0.2	46±5	22±4	30±1	43±3	0.8±0.1	2
	6	2.9±0.2	63±9	36±5	45±6	49±10	1.1±0.0	2
	7	2.7±0.5	68±20	30±5	42±10	55±12	1.5±0.5	2
	8	3.0±0.8	44±4	18±10	27±8	54±22	1.1±0.4	2
	9	3.1±1.0	32±0	12±4	18±2	53±23	1.0±0.5	2
**ES+/BMS-**	0	2.5±0.0	64±11	25±8	38±9	48±17	1.5±0.6	2

	1	2.7±0.0	79±18	34±13	49±15	52±2	1.3±0.0	2
	2	3.1±0.3	77±2	35±2	49±2	50±4	0.9±0.2	2
	3	3.3±0.2	67±5	31±4	43±1	37±1	0.5±0.1	2
	4	3.2	67	28	41	33	0.5	1
	5	3.2	66	33	44	37	0.6	1
	6	3.2	65	20	35	60	0.9	1
	7	3.2	69	23	38	64	1.0	1
	8	3.2	65	24	38	56	0.8	1
**ES+/BMS+**	0	1.6±0.5	76±19	38±19	51±19	35±7	6.6±6.7	3
	1	2.3±0.6	90±2	48±4	62±2	32±4	1.7±1.3	3
	2	2.6±0.4	89±11	42±8	57±8	40±14	1.2±0.4	3
	3	2.6±0.3	89±4	36±1	54±2	43±6	1.2±0.4	3
	4	2.6±0.4	94±13	45±2	62±5	41±7	1.2±0.3	3
	5	2.6±0.4	94±12	47±10	63±11	37±12	1.1±0.2	3
	6	3.1	79	37	51	53	0.9	1
**Slaughterhouse**	0	2.7±0.3	86±13	49±1	61±5	50±19	1.2±0.1	2
**(ES+/BMS+)**	1	2.9±0.2	100±8	53±1	69±4	57±28	1.1±0.4	2
	2	3.1±0.2	96±7	48±1	64±3	58±21	1.0±0.1	2
	3	3.2±0.2	87±4	47±16	60±12	62±12	0.9±0.0	2
	4	3.3±0.2	89±2	45±17	60±12	59±15	0.8±0.1	2
	5	3.3±0.2	87±4	45±14	59±11	59±16	0.8±0.1	2
	6	3.5	90	53	65	73	0.9	1
	7	3.5	89	50	63	72	0.8	1
	8	3.5	92	52	65	81	0.9	1

Supplementary Table 1: Hemodynamic parameters of the RCA cultures in the *ex vivo* vascular bioreactor. Data are presented as mean ± SD. D = diameter; SP = systolic pressure; DP = diastolic pressure; MAP = mean arterial pressure; Qpeak = flow; SSpeak = shear stress.

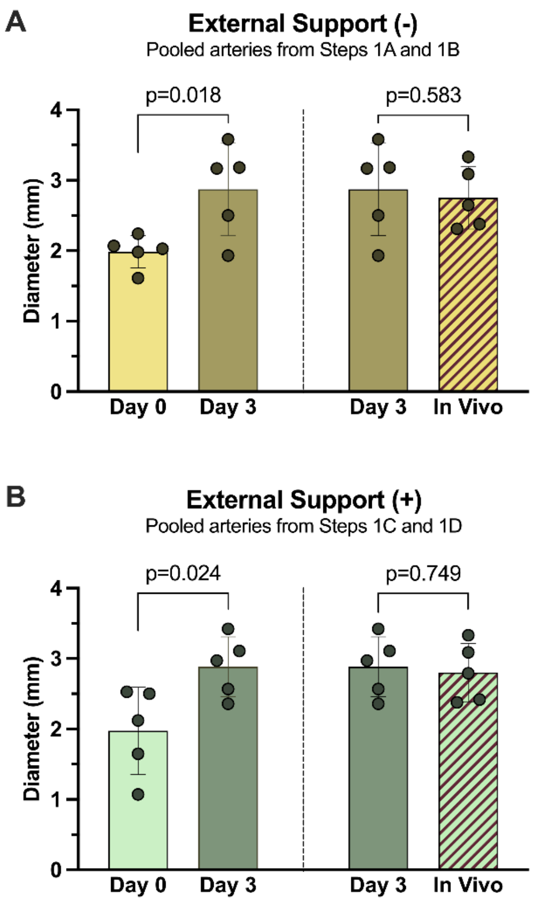

Supplemental Figure 1 *shows the in-culture vascular diameters at days 0 and 3, as well as the in vivo diameters. The data indicate that in culture, vascular diameters increase significantly, but return to in vivo values by day 3, both without (A) and with (B) external support. Data represented in A were pooled from step 1A and 1B, data represented in B were pooled from step 1C and 1D.*

Supplementary Table 2
In vivo coronary diameters of the in-house swine

Without external support (mm)	With external support (mm)
2.65	2.38
2.31	3.33
3.09	3.09
2.38	2.42
3.33	2.79

In vivo coronary diameters, as determined by in vivo quantitative coronary angiography, of the in-house swine used to optimize the VABIO settings.
